# Left atrial dysfunction in sickle cell anemia is associated with diffuse myocardial fibrosis, increased right ventricular pressure and reduced exercise capacity

**DOI:** 10.1038/s41598-020-58662-8

**Published:** 2020-02-04

**Authors:** Tarek Alsaied, Omar Niss, Justin T. Tretter, Adam W. Powell, Clifford Chin, Robert J. Fleck, James F. Cnota, Punam Malik, Charles T. Quinn, Sherif M. Nagueh, Michael D. Taylor, Wojciech M. Mazur

**Affiliations:** 1 Divisions of Cardiology, Cincinnati Children’s Hospital Medical Center, Cincinnati, OH USA; 2 Divisions of Hematology, Cincinnati Children’s Hospital Medical Center, Cincinnati, OH USA; 3 Department of Radiology at Cincinnati Children’s Hospital Medical Center, Cincinnati, OH USA; 4 Experimental Hematology and Cancer Biology, Cincinnati Children’s Hospital Medical Center, Cincinnati, OH USA; 5 Houston Methodist DeBakey Heart and Vascular Center Houston, Texas, USA; 6 The Christ Hospital Health Network Cincinnati, Ohio, USA

**Keywords:** Cardiovascular biology, Cardiovascular diseases

## Abstract

Increased extracellular volume (ECV) by CMR is a marker of interstitial myocardial fibrosis and is associated with diastolic dysfunction in sickle cell anemia (SCA). Left atrial (LA) dysfunction and stiffness contribute to the development of diastolic heart failure in other settings. We aimed to evaluate LA function and stiffness associations with ECV, tricuspid regurgitation jet velocity (TRV) and exercise abnormalities in SCA. In a prospective study, individuals with SCA underwent CMR, echocardiography and exercise test. ECV was measured using MOLLI sequence. Atrial strain was studied in the 4- and 2-chamber views. LA stiffness was calculated as the ratio of echocardiographic E/e’-to-LA reservoir strain. Twenty-four participants with SCA were included (median age 20 years). ECV was increased in participant with SCA compared to our lab normal values (mean 0.44 ± 0.08 vs 0.26 ± 0.02, P < 0.0001). Six (25%) had LA LGE. ECV positively correlated with LA stiffness (r = 0.45, p = 0.04). There was a negative correlation between LA stiffness and %predicted VO2 (r = −0.50, p = 0.04). LA stiffness was moderately associated with increased TRV (r = 0.55, p < 0.005). LA stiffness is associated with ECV, exercise impairment and increased TRV. This study sheds insights on the interaction between LA function, RV hypertension, and myocardial fibrosis in SCA.

## Introduction

Sickle cell anemia (SCA) affects approximately 1 in 700 African-Americans, and as many as 100,000 individuals in the United States^1^. Cardiac complications are important causes of morbidity and mortality in SCA^2,3^. Diastolic dysfunction (DD) and pulmonary hypertension are known cardiac complications of SCA and are independent risk factors for early mortality^4–6^. DD is associated with microscopic, interstitial myocardial fibrosis in SCA mice and with diffuse myocardial fibrosis, assessed by cardiac MRI (CMR) using extracellular volume (ECV), in humans with SCA^3,7,8^.

Left atrial (LA) function has not been studied before in patients with SCA. Noninvasive comprehensive evaluation of LA function is now possible using strain Doppler echocardiography with reasonable accuracy^9,10^. Increased LA stiffness in patients with DD is associated with the development of heart failure and exercise impairment although that has not been studied in SCA^9,11,12^.

Here we sought to evaluate LA function in patients with SCA and determine any associations between LA stiffness and ECV, tricuspid regurgitation jet velocity (TRV) and exercise abnormalities.

## Methods

### Participants and study design

Participants with SCA were enrolled in a prospective, longitudinal CMR study to characterize SCA-related cardiomyopathy. Participants underwent a CMR and an echocardiogram and an optional CPET. The main exclusion criteria were chronic transfusion therapy and glomerular filtration rate <60 mL/min/1.73 m^2^. The study was approved by the Institutional Review Board of Cincinnati Children’s Hospital. Informed consent was obtained from adults or parents of minor participants^13^. All the methods were performed in accordance with the relevant guidelines and regulations.

### CMR protocol and image analysis

CMR was performed on a 1.5 T scanner (Philips Ingenia, Best, Netherlands). ECV was measured from T1-maps acquired with a modified Look-Locker inversion recovery (MOLLI) sequence^8^. All planimetric and T1 analyses were done with Cvi42 (Circle Imaging; Alberta, Canada)^13^.

Participants, all of whom had abnormally increased ECV, were sub-classified by degree of elevation of ECV into two groups: first group (0.33–0.44) and second group (>0.44). The cutoff for ECV of 0.44 was the mean value for ECV in the entire SCA study population^8^. Baseline laboratory testing was obtained at the time of CMR including hemoglobin and N-terminal pro b-type natriuretic peptide (NT-proBNP).

### Echocardiographic studies

Transthoracic echocardiography was performed with a Philips iE-33 system (Philips Electronics; Andover, MA). Measurements were analyzed using Syngo Dynamics (Siemens Healthcare, Germany). Pulsed-wave Doppler was used to measure mitral and tricuspid inflow peak velocity at early (E) and late filling (A) between the leaflet tips. Tissue Doppler imaging was used to determine mitral and tricuspid valve annular velocities in early (e’) and late diastole (a’) at both the septal and lateral annulus. Continuous-wave Doppler sampling of the peak TRV was used from parasternal and apical windows^8^. Increasing color gain and decreasing the color Doppler Nyquist limit were used as needed to clarify the tricuspid regurgitation jet and obtain an adequate Doppler wave form. Agitated saline injection was also used as needed. The highest TRV was recorded.

### LA function

**Figure 1:**
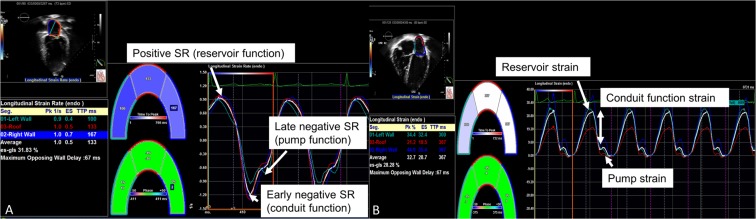
Atrial strain and strain rate by echocardiogram. (**A**) represent atrial strain curves measured over the cardiac cycle. The white curve represents average atrial strain curve. (**B**) represents atrial strain rate curve.

LA function was studied using two dimensional speckle tracking imaging^14,15^. Offline and blinded analysis to clinical outcomes was performed using TOMTEC software. The apical four-chamber and two chamber views were optimized for visualization of the LA. Patients with inadequate image quality were excluded. LA ejection fraction and fractional area change were calculated from echocardiography^16^. LA areas and volumes were derived in the apical four-chamber and two-chamber views using two-dimensional (Simpson’s method) echocardiography. LA ejection fraction was calculated as = [(maximal LA volume in ventricular systole just before mitral valve opening − minimal LA volume after mitral valve closure)/ maximal LA volume in ventricular systole just before mitral valve opening]^16^. The strain measurements were performed using the QRS complex (R-R gating) as the initiation of the strain calculation. There are two peaks in the strain curve. The first peak corresponds to reservoir function (first peak between R wave and T wave) and the second to atrial contractile function (starting on the P wave); the difference between reservoir strain and atrial contractile strain values reflects conduit function^15^. Positive global strain rate at the beginning of left ventricular systole reflects reservoir function. Early negative diastolic strain rate reflects conduit function while late diastolic global strain rate reflects pump function (Fig. 1)^14,15^. The E/e′ ratio (average e′) was also used in conjunction with the reservoir function strain to derive a noninvasive dimensionless parameter of LA stiffness^9^.

#### CPET

A maximal cardiopulmonary exercise was performed using an electronically braked cycle ergometer (Corival Load Cycle 400)^13^. Gas exchange at rest, during exercise, and during recovery was analyzed to determine measures of oxygen consumption (VO_2_), carbon dioxide production (VCO_2_), minute ventilation (VE), and VE/VCO_2_ slope^17–19^. Because peak VO_2_ is influenced by age, sex, and body weight, %predicted VO_2_ was used to account for these variables in our study^20^.

Reduced exercise capacity was defined as %predicted VO_2_ <80%. Mild impairment of exercise capacity was defined as %predicted VO_2_ 60–80% while moderate-to-severe impairment was defined as %predicted VO_2_ <60%^21^.

### Atrial late gadolinium enhancement (LGE)

**Figure 2:**
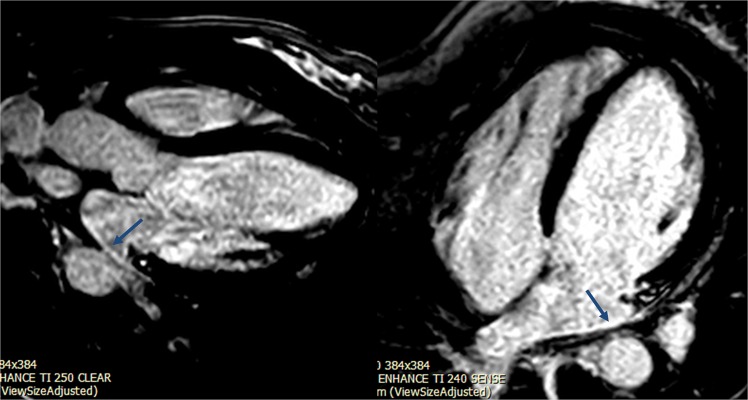
Left atrial late gadolinium enhancement in a patient with SCA in four chamber and three chamber views (blue arrows).

LGE imaging was performed with a standard phase-sensitive inversion recovery sequence protocol 10 minutes after injection with gadolinium-diethylenetriamine penta-acetic acid^8^. LA LGE was assessed in the two chamber, three chamber and four chamber orientation (Fig. 2). The short axis stack did not extend to include the entire left atrium and thus quantification of fibrosis (Utah score) was not feasible^16^. Two cardiologists reviewed the images and agreed on the presence of LA LGE.

### Statistical analysis

A student *t*-test or Mann-Whitney *U* test was used to compare 2 groups of continuous parametric or non-parametric variables, respectively, or Fisher’s exact test for categorical variables. Associations between normally distributed variables were calculated using the Pearson correlation coefficient. All P-values were two-tailed and differences were considered significant when P < 0.05. Statistical analyses were performed using JMP®, Version 12 from SAS Institute Inc. (Cary, NC).

## Results

### Patient characteristics and exercise performance

Twenty-six children and adults with SCA (homozygous HbSS) were enrolled, and 24 had adequate images to evaluate LA function by echocardiography and thus were included in the analysis. Twenty patients completed CPET. The median age was 20 years (range 7–61 years) (Table 1). ECV was increased in all SCA patients as compared to our lab normal control values (0.44 ± 0.08 vs 0.26 ± 0.02, P < 0.0001)^8^. Twelve patients had ECV > 0.44. There was no left or right ventricular LGE.

**Table 1:** Baseline clinical and laboratory characteristics of study participants.

Characteristic	Value
Age (yr)	23.0 ± 10.4
BSA (m^2^)	1.69 ± 0.3
Female, n (%)	13 (54)
Receiving hydroxyurea, n (%)	19 (79)
Baseline Heart rate (bpm)	74 ± 10
Systolic blood pressure (mmHg)	118 ± 11
Diastolic blood pressure (mmHg)	67 ± 8
White blood cell count (10^3^/mm^3^)	9.6 ± 3.6
Hemoglobin (g/dL)	9.9 ± 1.4
Mean corpuscular volume (fL)	94 ± 19
Reticulocyte count (%)	8.1 ± 5.2
Platelet count (10^3^/mm^3^)	343 ± 98
Bilirubin (mg/dL)	2.4 ± 1.5
AST (unit/L)	48 ± 29
LDH (unit/L)	550 ± 271
Plasma free hemoglobin (mg/dL)	26 (11–105)
Creatinine (mg/dL)	0.57 ± 0.17
Cystatin C (mg/L)	0.64 ± 0.13
GFR (mL/min/1.73 m^2^)	145 ± 38
NT-proBNP (pg/mL)	55 (23–150)
Native T1 (ms)	1005 ± 67
ECV	0.44 ± 0.08
% Predicted maximum oxygen consumption	57 ± 12
VE/VCO_2_ slope at maximum exercise	30 ± 7

The values are reported as mean ± standard deviation or median (interquartile range). AST: aspartate aminotransferase, ECV: extracellular volume, GFR: glomerular filtration rate, LDH: lactate dehydrogenase, min: minute, VO2: oxygen consumption, VCO2: carbon dioxide production, yr: year.

As previously reported, patients with SCA had significant exercise impairment (mean VO_2_ = 21.6 ± 6.1 ml/kg/min and mean %predicted VO_2_ = 57.0 ± 12.4%)^13^. Of the 18 patients who reached maximum exercise, 17 (94%) had reduced exercise capacity defined as %predicted VO_2_ <80%; of whom 5 (29%) had mild impairment (%predicted VO_2_ 60–80%) and 12 (67%) had moderate-to-severe impairment (%predicted VO_2_ <60%). Hemoglobin positively correlated with exercise capacity (r = 0.45, p = 0.04).

Patients in the ECV >0.44 group had lower peak work rate on CPET (Table 2).

**Table 2:** Clinical, laboratory, and exercise parameters of study participants based on ECV.

Characteristic	ECV ≤0.44 (n = 12)	ECV >0.44 (n = 12)	*p Value*
Age (yr)	23.0 ± 11.8	23.1 ± 9.2	0.98
Body surface area (m^2^)	1.68 ± 0.33	1.61 ± 0.42	0.64
Hemoglobin (g/dL)	10.3 ± 1.5	9.4 ± 1.3	0.14
Plasma free hemoglobin (mg/dL)	99 ± 137	24 ± 28	0.21
Hemoglobin F (%)	17 ± 13	19 ± 13	0.73
Heart rate at exercise cessation (BPM)	174 ± 18	162 ± 16	0.15
Peak work rate (Watt)	369 ± 332	141 ± 108	**0.04**
Respiratory exchange rate	1.4 ± 0.1	1.3 ± 0.2	0.17
VO_2_ at maximum exercise (mL/kg/min)	22 ± 2	20 ± 2	0.47
% predicted VO_2_ at maximum exercise	57 ± 13	54 ± 10	0.39
VE/VCO_2_ slope at maximum exercise	30 ± 7	32 ± 8	0.68
Moderate-to-severe exercise impairment, n (%)	8/12 (67)	4/6 (67)	0.98
VE/VO_2_ at maximum exercise	42 ± 11	40 ± 6	0.65
NT-Pro BNP (pg/mL)	31 (19–72)	149 (38–169)	**0.008**
FEV1 (%)	82 ± 12	82 ± 18	0.98
FVC (%)	87 ± 13	89 ± 14	0.76
FEV1/FVC (%)	93 ± 11	92 ± 7	0.10

ECV: extracellular volume, FEV1: forced expiratory volume in the first second, FVC: forced vital capacity, RLD: Restrictive lung disease, VO2: oxygen consumption, VCO2: carbon dioxide production, Yr: Year.

#### Atrial function and ECV

**Figure 3:**
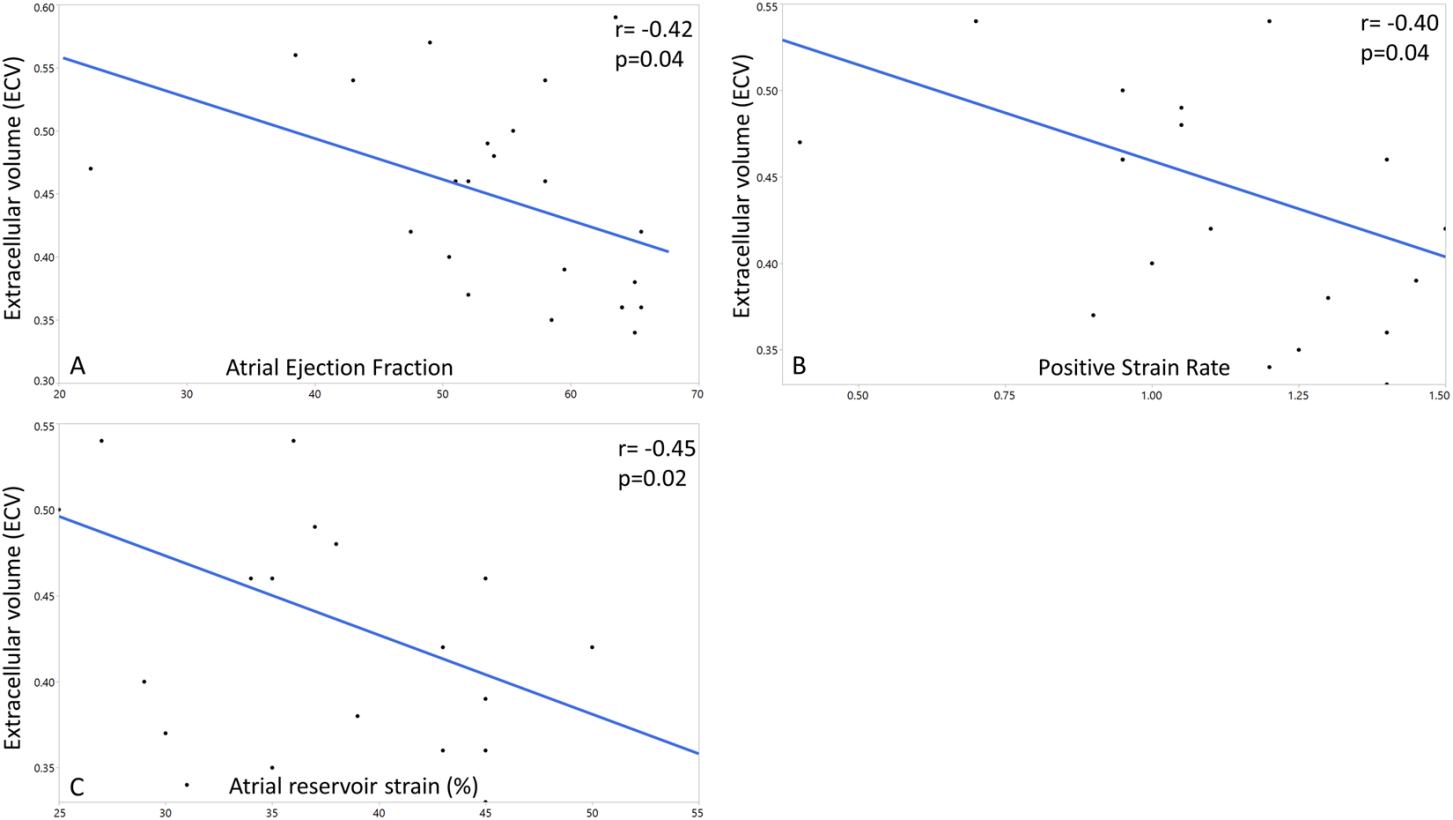
Left ventricular extracellular volume (ECV) negatively associated with atrial ejection fraction (**A**), positive strain rate (**B**) and atrial reservoir strain (**C**).

ECV negatively correlated with atrial ejection fraction (r = −0.42, p = 0.04). LA reservoir strain and positive global strain rate negatively correlated with ECV (r = −0.45, p = 0.02 and r = −0.40, p = 0.04). (Fig. 3) ECV was positively associated with LA stiffness (r = 0.45, p = 0.04). Patients in the ECV > 0.44 group had lower atrial ejection fraction, reservoir strain and positive strain rate as well as higher LA stiffness (Table 3). The ECV > 0.44 group had increased NT-Pro BNP and LA stiffness positively correlated with NT-pro BNP (r = 0.55, p = 0.003).

**Table 3:** Echocardiographic and cardiac MRI parameters of study participants based on ECV.

Characteristic	ECV ≤ 0.44 (n = 12)	ECV > 0.44 (n = 12)	*p Value*
**Echocardiographic Measures**			
TRV, m/s	2.5 ± 0.3	2.5 ± 0.7	0.96
E/A ratio	2.0 ± 0.60.6	1.8 ± 0.6	0.30
Lateral e′ (cm/s)	14 ± 4	15 ± 4	0.33
Lateral E/e′ ratio	7 ± 2	8 ± 3	0.21
Septal e′(cm/s)	10 ± 2	11 ± 1	0.21
Septal E/e′ ratio	9 ± 2	9 ± 2	0.85
Lateral a’ (cm/s)	8 ± 2	7 ± 2	0.64
Septal a’ (cm/s)	8 ± 2	7 ± 2	0.69
Heart rate	76 ± 12	77 ± 13	0.82
Systolic blood pressure	126 ± 12	121 ± 9	0.33
Distolic blood pressure	65 ± 9	60 ± 8	0.19
Atrial end diastolic volume(mL/m^2^)	30 ± 10	29 ± 14	0.85
Atrial end systolic volume (mL/m^2^)	70 ± 18	62 ± 32	0.40
Atrial ejection fraction (%)	60 ± 7	49 ± 11	**0.02**
Atrial global longitudinal strain (%)	32 ± 8	26 ± 9	0.08
Atrial fractional area change (%)	44 ± 7	37 ± 9	0.07
Atrial reservoir strain (%)	40 ± 7	33 ± 10	**0.04**
Atrial conduit strain	31 ± 7	26 ± 8	0.17
Atrial pump strain	9 ± 4	7 ± 2	0.17
Atrial positive strain rate	1.3 ± 0.2	1.0 ± 0.3	**0.01**
Atrial early negative strain rate	−1.4 ± 0.5	−1.2 ± 0.4	0.45
Atrial late negative strain rate	−0.8 ± 0.2	−0.7 ± 0.4	0.56
Left atrial stiffness	0.18 ± 0.05	0.26 ± 0.10	**0.03**
**Cardiac MRI Measures**			
LAVi, mL/m^2^	50 ± 13	55 ± 12	0.39
LVEDVi, mL/m^2^	101 ± 21	119 ± 22	0.06
LVESVi, mL/m^2^	38 ± 10	48 ± 15	0.08
LVSVi, mL/m^2^	61 ± 13	71 ± 12	0.08
LVEF, %	62 ± 4	60 ± 6	0.31
RV cardiac index, L/min per m^2^	62 ± 15	67 ± 20	0.50
RVEDVi, mL/m^2^	105 ± 23	119 ± 28	0.18
RVEDVi, mL/m^2^	105 ± 23	119 ± 28	0.18
RVESVi, mL/m^2^	43 ± 4	52 ± 5	0.15
RVSVi, mL/m^2^	62 ± 12	68 ± 13	0.31
RVEF, %	59 ± 5	57 ± 5	0.25
LV cardiac index, L/min per m^2^	4.6 ± 1.0	5.3 ± 1.4	0.18
RV cardiac index, L/min per m^2^	4.6 ± 1.0	4.7 ± 2.0	0.84

#### Atrial function and exercise capacity

**Figure 4:**
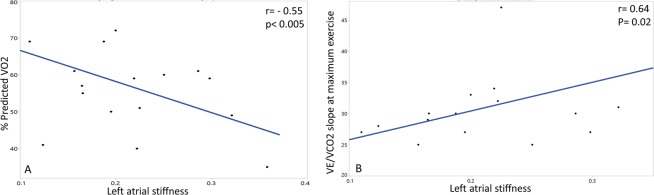
Left atrial stiffness negatively associated with percent predicted VO_2_ (**A**) and positively associated with VE/VCO_2_ slope at maximum exercise.

There was a negative correlation between LA stiffness and %predicted VO_2_ (r = −0.50, p = 0.04) and a positive correlation between LA stiffness and VE/VCO_2_ slope at maximum exercise (r = 0.64, p = 0.02). (Fig. 4) Patients with moderate to severe exercise impairment had significantly higher LA stiffness as compared to patients with mild exercise impairment or normal exercise capacity (0.26 ± 0.10 vs 0.18 ± 0.05, p = 0.004).

#### LA function and tricuspid regurgitation jet velocity

**Figure 5:**
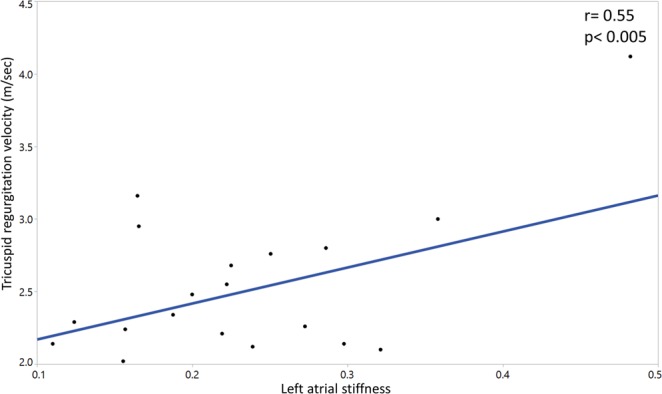
Left atrial stiffness correlates positively with tricuspid regurgitation velocity.

There was a significant positive association of the LA stiffness and TRV (r = 0.55, p < 0.005). (Fig. 5)

#### Atrial function by echo and atrial volume by CMR

As expected, there was a strong correlation between CMR derived LA maximum volume and atrial end-diastolic (p = 0.008) and end systolic volumes by echocardiogram (p = 0.004). However, there was no association between atrial volume by CMR or echocardiogram and atrial function measures by echocardiogram. Absolute and indexed atrial volumes by CMR and echocardiogram did not correlate with exercise capacity or ECV.

#### Atrial late gadolinium enhancement

Six of the 24 participants (25%) had LA LGE (Fig. 2). When participants were dichotomized based on the presence of LA LGE we did not find a significant difference in exercise capacity or LA functional measures.

## Discussion

We have shown that diffuse myocardial fibrosis, defined by increased ECV, in individuals with SCA is associated with LA dysfunction. LA stiffness was associated with both reduced exercise capacity and increased TRV, which is a predictor for early mortality in adults with SCA^6,22^. We also observed macroscopic LA fibrosis by LGE in 6 of the 24 participants. To our knowledge, this is the first report of impaired LA function in SCA and its association with diffuse myocardial fibrosis, exercise capacity and TRV.

LA enlargement is a predictor of adverse cardiovascular outcomes in other diseases^23^. In patients with SCA, chronic LA enlargement is common and is likely due to a combination of DD and the chronic increase in cardiac output to meet the oxygen demand^8,13,24^. In our study we found that the atrial function measures rather than the size of the LA correlated with ventricular fibrosis, TRV and exercise capacity which is likely due to the multifactorial etiology of LA enlargement. LA enlargement may result in LA mechanical and electrical remodeling^25^. Progressive atrial dilation may eventually reach a threshold fiber length where atrial shortening and contractility will begin to decline. In addition, LA enlargement is associated with an increased risk for arrhythmia^26^. With recent advances in non-invasive technologies, reliable assessment of the LA function is now possible using speckle tracking imaging^25^.

We found that with increasing left ventricular ECV (i.e., increasing ventricular interstitial fibrosis), the LA stiffness increases and atrial ejection fraction and reservoir function decrease. LA and the LV have a dynamic interaction which is described in many disease states^27,28^. Our previous work showed an association between ECV and DD in SCA^8,13^. It is also possible that chronic volume overload contributes to ventricular and atrial remodeling and impaired function^29^. Previous animal studies showed myocardial fibrosis and LA enlargement in SCA mice but not in mice with iron deficiency anemia suggesting that anemia and volume overload are not the main mechanism for LA enlargement and increased ECV in SCA^30^.

The association between LA stiffness and TRV in this study is notable. Previous studies suggested that LA function is an important predictor of pulmonary hypertension in cases of LA volume overload such as mitral regurgitation^31^. In patients with LV diastolic dysfunction, LA stiffness is also associated with the development of symptomatic heart failure and pulmonary hypertension^9^. In SCA, both increased preload, due to chronic increased cardiac output, and afterload, due to left ventricular DD, result in atrial stiffness and may contribute to post-capillary pulmonary hypertension in this population^4,24,32,33^. Thus LA stiffness may be a significant marker of myocardial disease in SCA.

Impairment of exercise tolerance is common in children and young adults with SCA, but the degree to which cardiopulmonary disease contribute to this impairment is not known^18,34,35^. Our study demonstrates a negative association between LA stiffness and exercise capacity as measured by %predicted VO_2_ and with ventilation efficiency measured by VE/VCO_2_ slope. This suggests that increased LA stiffness may be associated with lower cardiac output or with increased pulmonary capillary wedge pressure and increased pulmonary congestion resulting in decreased exercise capacity^4,32^. LA stiffness index reflects the interaction between left ventricular compliance and LA reservoir function^36^. Interestingly, LA volume by CMR and echocardiogram did not correlate with ECV, TRV or exercise capacity in our study, likely because of the confounding effect of volume overload in SCA. The increase in LA stiffness and the decrease in LA compliance may be an early precursor for the development of elevated right ventricular pressure in SCA individuals with DD^37,38^. Further studies are needed to elucidate that.

We found LA LGE in 6 participants. LA LGE is linked to the development of atrial arrhythmia in other patient populations^39^. Dysrhythmias, including atrial arrhythmias, have been linked to premature death and are poorly understood in SCA^2,40^. In our study LA LGE did not correlate with LA strain or with any outcomes and this could be due to the small sample size and the technical limitations of our LA LGE technique. The LA LGE findings in our study should be interpreted with care, because our CMR was not originally obtained to evaluate for atrial LGE, which was an incidental finding. Many technical improvements are necessary to accurately assess LGE in the thin LA wall^41^. The significance of these findings will be the focus of future studies.

Our study has several limitations. First, this is a relatively small sample that may limit the interpretation of the correlations between variables and prevent the use of multivariate analysis. Despite the small sample size, the findings of this study are novel and can be the basis of larger confirmatory and mechanistic studies. Second, this study included a wide age range (6–60) years. As SCA is a life-long disease, including a wide age range was felt to be appropriate and necessary for an initial study, but multiple age subgroups were too small for meaningful analysis.

Third, our original study design was focused on ventricular evaluation. Short axis post contrast views of the LA were not acquired and quantitative assessment of LA LGE was not possible. In addition, our study does not address if LA stiffness is secondary to LV fibrosis-related DD, LA fibrosis, or both.

**Figure 6:**
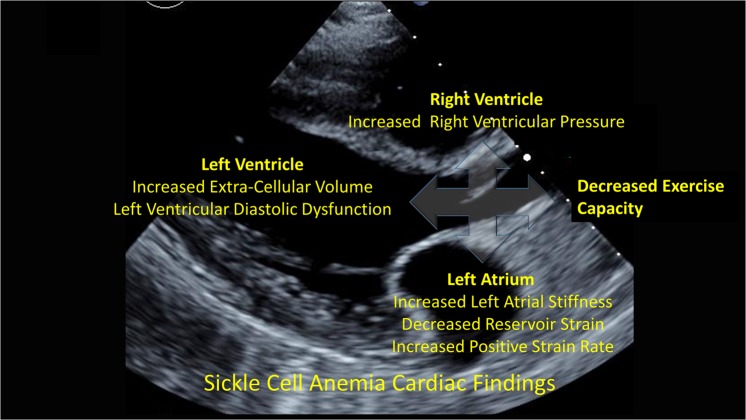
Summary of cardiac involvement in sickle cell anemia.

In summary, we have shown that LA stiffness is associated with elevated TRV and poor exercise capacity in children and adults with SCA. LA dysfunction is also associated with ventricular ECV, suggesting that LV diffuse interstitial myocardial fibrosis may lead to impaired LA function and subsequently to elevated pulmonary pressures (Fig. 6). The therapeutic targeting of both atrial stiffness and ventricular fibrosis to potentially ameliorate cardiac complications and improve outcomes in SCA are needed.
