# Author Correction: Left atrial dysfunction in sickle cell anemia is associated with diffuse myocardial fibrosis, increased right ventricular pressure and reduced exercise capacity

**DOI:** 10.1038/s41598-020-61554-6

**Published:** 2020-03-12

**Authors:** Tarek Alsaied, Omar Niss, Justin T. Tretter, Adam W. Powell, Clifford Chin, Robert J. Fleck, James F. Cnota, Punam Malik, Charles T. Quinn, Sherif F. Nagueh, Michael D. Taylor, Wojciech M. Mazur

**Affiliations:** 1 Divisions of Cardiology, Cincinnati Children’s Hospital Medical Center, Cincinnati, OH USA; 2 Divisions of Hematology, Cincinnati Children’s Hospital Medical Center, Cincinnati, OH USA; 3 Department of Radiology at Cincinnati Children’s Hospital Medical Center, Cincinnati, OH USA; 4 Experimental Hematology and Cancer Biology, Cincinnati Children’s Hospital Medical Center, Cincinnati, OH USA; 5 Houston Methodist DeBakey Heart and Vascular Center Houston, Texas, USA; 6 The Christ Hospital Health Network Cincinnati, Ohio, USA

Correction to: *Scientific Reports* 10.1038/s41598-020-58662-8, published online 04 February 2020

The original version of this Article contained a typographical error in the spelling of the author Sherif F. Nagueh, which was incorrectly given as Sherif M. Nagueh.

As a result, in the Author Contributions,

“Sherif M. Nagueh helped in the study design, analyzed echo data and edited the manuscript.”

now reads:

“Sherif F. Nagueh helped in the study design, analyzed echo data and edited the manuscript.”

This error has now been corrected in the PDF and HTML versions of the Article.

